# High Milling Time Influence on the Phase Stability and Electrical Properties of Fe_50_Mn_35_Sn_15_ Heusler Alloy Obtained by Mechanical Alloying

**DOI:** 10.3390/ma17174355

**Published:** 2024-09-03

**Authors:** Florin Popa, Traian Florin Marinca, Niculina Argentina Sechel, Dan Ioan Frunză, Ionel Chicinaș

**Affiliations:** Department of Materials Science and Engineering, Technical University of Cluj-Napoca, 103-105 Muncii Avenue, 400641 Cluj-Napoca, Romania; traian.marinca@stm.utcluj.ro (T.F.M.); niculina.sechel@stm.utcluj.ro (N.A.S.); dan.frunza@ipm.utcluj.ro (D.I.F.); ionel.chicinas@stm.utcluj.ro (I.C.)

**Keywords:** Heusler alloys, mechanical milling, nanocrystalline, lattice strain, electrical resistivity

## Abstract

Fe_50_Mn_35_Sn_15_ Heusler alloy, obtained by mechanical alloying, was subjected to larger milling times in the range of 30–50 h to study the phase stability and morphology. X-ray diffraction studies have shown that the milled samples crystallise in a disordered A_2_ structure. The A_2_ structure was found to be stable in the milling range studied, contrary to the computation studies performed on this composition. Using Rietveld refinements, the lattice parameter, mean crystallite size, and lattice strain were computed. The nature of the obtained phases by milling was found to be nanocrystalline with values below 50 nm. A linear increase rate of 0.00713 (h^−1^) was computed for lattice strain as the milling time increased. As the milling time increases, the lattice parameter of the cubic Heusler was found to have a decreasing behaviour, reaching 2.9517 Å at 50 h of milling. The morphological and elemental distribution—characterised by scanning electron microscopy and energy-dispersive X-ray spectroscopy—evidenced Mn and Sn phase clustering. As the milling time increased, the morphology of the sample was found to change. The Mn and Sn cluster size was quantified by elemental line profile. Electrical resistivity evolution with milling time was analysed, showing a peak for 40 h of milling time.

## 1. Introduction

In the quest for novel material for practical applications, it is important that easily tuneable properties be obtained using as-simple-as-possible methods and without involving too many steps. In electronics, complex components are obtained by successive treatments of Si wafers, and a similar approach is desired for sensor production. One class of materials that can fit this description are Heusler alloys. This is possible since the properties of the Heusler alloys can be tailored by composition and valence electrons [1]. To make these alloys even more attractive, a coupling is envisaged between different properties such as magnetic, magnetoelastic, thermoelectric, topological, or superconducting properties [2]. By combining the occurrence of different magnetic properties (ferromagnetism, nonmagnetic, insulating, and antiferromagnetism), devices for spintronic applications can be developed [3]. These features occur naturally from the complex structure of the Heusler alloys and element combinations. The Heusler alloys have a chemical formula X_2_YZ, where each of the composing elements is located in a specific position [4,5]. The structure of a Heusler alloy is a face-centred cubic, but to accommodate this large number of different atoms, it is actually formed from four interpenetrating face-centred cubic lattices. If the atoms are located in the (¼, ¼, ¼) and (¾, ¾, ¾) Wyckoff position for the X atoms, (½, ½, ½) Wyckoff position for the Y atoms, and (0, 0, 0) Wyckoff position for the Z atoms, the structure is ordered (namely an L_21_ structure) [4,6]. However, this order is not always achieved, and several disorder degrees can occur [7,8,9]. The first disorder degree can be between the Y and Z atoms, and this structure is called B_2_. The degree of disorder can be even greater, and all the atoms can randomly occupy a site, leading to the A_2_ structure [7,8,9]. To be complete, even an inverse-ordered structure can be formed, where the X atoms go to the (0, 0, 0) and (¼, ¼, ¼) sites, the Y atoms go to (½, ½, ½) site, and the Z atoms go to (¾, ¾, ¾) site [10]. Speaking of composition, this ternary alloy class is formed from d metals for the X and Y atoms, and Z is a p main group element [11,12].

Among the Heusler alloys, Fe_2_XY alloy present scientific interest since it is believed to be a good candidate for a magnetic shape memory alloy [13,14]. In this family of alloys, it was found that if V and Al are used alongside Fe, semiconductor behaviour is obtained. The electron concentrations can be controlled by doping, with all sites being able to accommodate doping elements [15]. If Al is replaced with Ge, the Fe_2_VGe alloy has an interesting behaviour since its lattice parameters are dependent on induced defects [16]. In the Fe_2_MnZ alloys, the magnetic moment is dependent on the lattice parameter values [17]. Regarding the structure of the Fe_2_XY Heusler alloy, a large portion of the studies carried out indicated that by arc melting, the obtained structure is hexagonal [18]. The indexed hexagonal structure is a DO_19_ structure [19,20]. Other studies have shown that the Fe_2_MnSn Heusler alloy cannot be obtained as a single-phase but rather a three-phase alloy: Sn solid solution, Fe solid solution, and Fe_3_Sn_2_ compound [21]. If we discuss the off-stoichiometric alloy case, for the Fe-based Heusler alloys, it was found that the transport properties are enhanced [22].

This overview of Fe_2_MnSn Heusler alloys indicates that the usual way of obtaining Heusler alloys—by arc melting and high-temperature, long-time annealing—is not suitable. Therefore, alternatives are searched, and one proposed method was arc melting followed by milling [23], and it was found that mechanical milling can reduce the amount of hexagonal phase. Following these studies, we propose obtaining the Fe_50_Mn_35_Sn_15_ Heusler alloy directly via mechanical alloying from elemental powder, using long milling times. Applying the solid-state synthesis for Heusler alloys showed that Heusler alloys can be obtained but only in a disordered nanocrystalline state or as amorphous alloys [24,25,26,27].

The mechanical alloying method is an out-of-equilibrium synthesis method allowing for the alloy to be obtained at room temperature with high amounts of internal stresses and a nanocrystalline microstructure [28,29,30]. The process can be briefly described as a repetitive event of fracturing and welding the processed powders through the combined action of several balls sealed in vials and forced by rotational forces to collide. At each collision event, a powder quantity is trapped between the balls and a fraction of the kinetic energy is transferred from the balls to the powders. In this process, the elemental powders are uniformised in terms of chemical distribution and, finally, a reaction is triggered, thus forming a new alloy [31,32]

In this paper, we studied the influence of long milling times over the phase stability of the Fe_50_Mn_35_Sn_15_ Heusler alloy. The phases present in the milled powder are quantified using the Rietveld method, and lattice strain and mean crystallite size are discussed. Particle size modification via long-time milling is analysed and correlated with elemental phase distribution. In the end, the electrical resistivity of the milled sample is measured and analysed.

## 2. Materials and Methods

Using elemental powders of Fe (NC 100.24 (Höganäs, Halmstad), Mn ((Alpha Aesar, Haverhill, MA, USA), −325 mesh, 99.3% metal basis), and Sn ((Alpha Aesar, Haverhill, MA, USA), −325 mesh, 99.8% metal basis), the Fe_50_Mn_35_Sn_15_ Heusler alloy was obtained in a planetary ball mill. The used mill was a Pulveristte 6 Fritch model (Frisch, Idar-Oberstein, Germany), operated at 350 rpm and containing a 250 mL stainless steel vial. The elemental powder mixture was spatially homogenised for 15 min and transferred to the vial filled with 50 hardened steel balls with 14 mm diameters. To avoid oxidation, Ar gas was introduced in the vials. The milling was conducted for 30 to 50 h, with sampling being performed at selected intervals.

The structure and the phase were studied by X-ray diffraction using an INEL Equinox 3000 diffractometer (INEL, Artenay, France), operating with the CoKα radiation, λ = 1.79026 Å in the angular range of 20–110 degrees, in reflection mode. From the X-ray diffraction patterns, using the Rietveld method, the phases and lattice parameters were investigated [33,34]. The software used for performing the Rietveld fitting was Winplotr software (version April 2023) [35,36]. The internal stresses and mean crystallite size were computed using the full-width half-maximum of the recorded peaks following the procedure given by the Williamson–Hall method [37,38].

Morphology studies were given by analysing the images recorded by scanning electron microscopy with a JEOL JSM 5600LV microscope (JEOL, Tokyo, Japan). For selected areas, energy-dispersive X-ray spectroscopy (EDX) analysis was performed using an Oxford Instruments detector (UltimMAX65), using the Aztec software, version 4.2 (High Wycombe, UK).

Particle size distribution was analysed for the milled samples using an Analysette NanoTec 22 (Frisch, Idar-Oberstein, Germany) laser particle sizer in the particle size range of 0.1–1000 µm.

Electrical resistivity measurements were performed using a four-probe setup connected to a USB6009 acquisition board from National Instruments (Austin, TX, USA). For the electrical resistivity measurements, the samples were manually compacted into cylindrical shapes at 700 MPa.

## 3. Results

**Figure 1:**
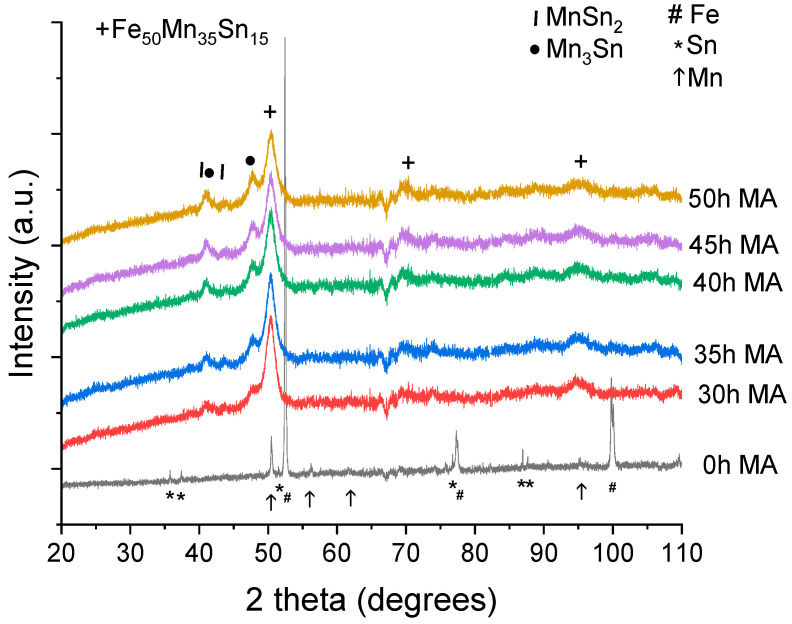
X-ray diffraction patterns for the Fe_50_Mn_35_Sn_15_ Heusler alloy samples milled for 30 to 50 h.

The X-ray diffraction patterns for the samples milled from 30 to 50 h are presented in Figure 1. To highlight the modifications induced by milling, the diffraction pattern of the initial elemental powder mixture is shown. From Figure 1, it is clear that a reaction occurred upon milling since the peak position of the initial elemental mixture is different for the sample milled at 30 h and beyond. Upon indexing the diffraction patterns for the milled samples, it is found that the main phase is a cubic Fe_50_Mn_35_Sn_15_ Heusler phase, completely disordered (the A_2_ structure). Alongside this cubic Heusler phase, two additional Mn phases are found: MnSn_2_ and Mn_3_Sn. Compared with other studies, where a hexagonal structure is formed [18,23], this is surprising. On the other hand, in the Fe_2_MnAl system, if milling is added to an arc-melted sample, the A_2_ structure is obtained [39].

**Figure 2:**
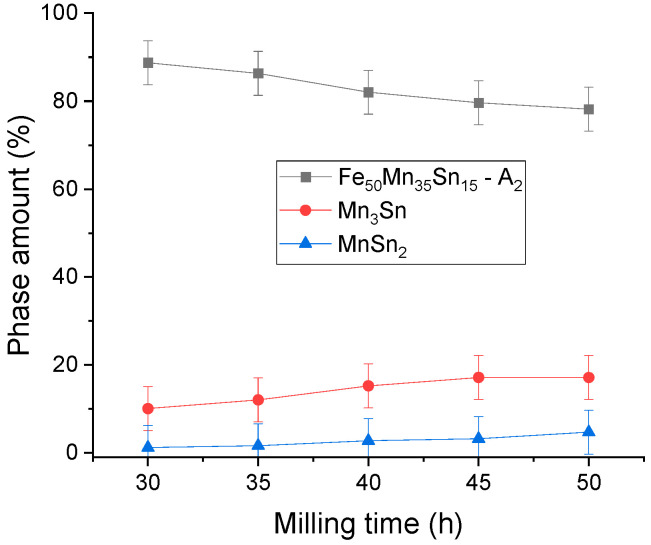
Phase evolution for the milled Fe_50_Mn_35_Sn_15_ samples.

In the milling time range of 30–50 h, visible changes in the position and number of the diffraction peaks are not seen, meaning that the phases are stable upon milling in this range. Using the Rietveld refinement procedure, the number of phases was computed for each milling time, and the results are plotted in Figure 2.

Analysing the phase weight evolution, a decreasing tendency for the Heusler phase is recorded, and at the same time, a slight increase in the Mn phase amount is visible.

**Figure 3:**
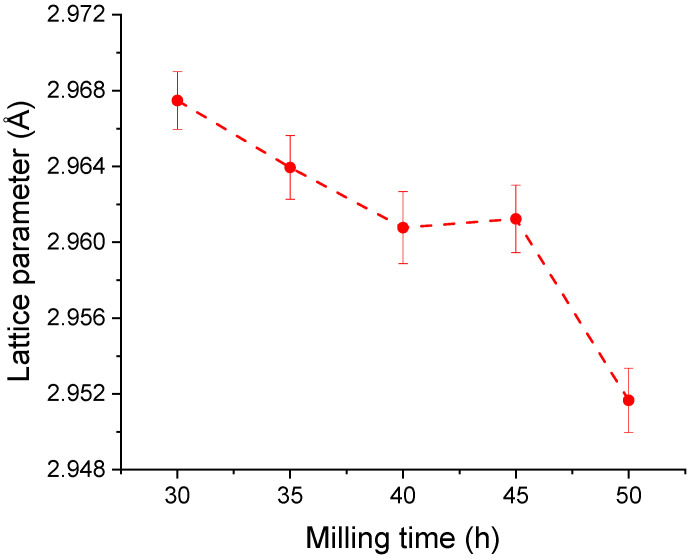
Lattice parameter of the Fe_50_Mn_35_Sn_15_ Heusler alloy milled for times ranging from 30 to 50 h.

Considering the phase’s relative stability, other structural parameters were analysed versus milling time to evaluate the influence of the prolonged milling time on the Fe_50_Mn_35_Sn_15_ Heusler alloy. Firstly, the lattice parameter was computed, and the obtained values are given in Figure 3.

The lattice parameter of the Fe_50_Mn_35_Sn_15_ Heusler alloy has a continuous decrease, almost linear with the milling time. The computed values vary from 2.9675 Å at 30 h of milling to 2.9517 at 50 h of milling, representing a shrinkage in the cell of 1.5%. The elemental cell shrinkage is related to the disordered alloy, obtained by milling and by the formation of vacancies. The lattice parameter shrinkage was found in other milled compounds [40,41] and can be related to the internal stresses induced by milling or element diffusion [42] or, in our case, by phase shift.

**Figure 4:**
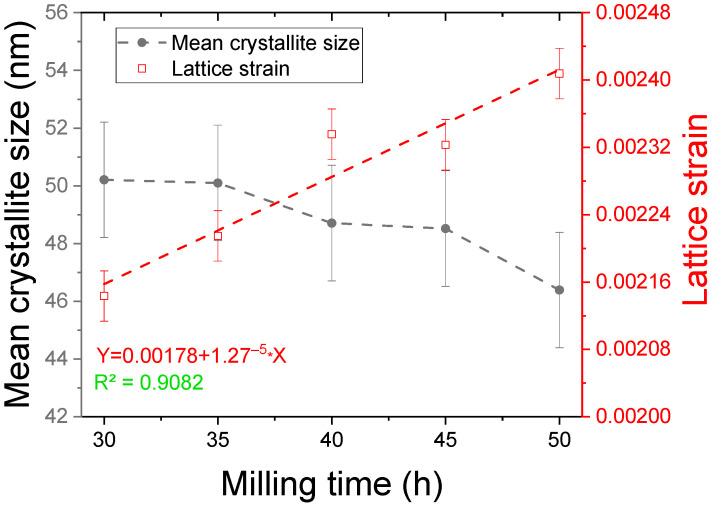
Mean crystallite size and lattice strain variation with the milling time for the Fe_50_Mn_35_Sn_15_ Heusler alloy milled for 30 to 50 h.

Other computed parameters were the mean crystallite size and lattice strain; for these parameters, the evolution with the milling time is presented in Figure 4.

The mean crystallite size has a steady decreasing evolution with milling time, from a value of 50 ± 2 nm for the 30 h milled sample to 46 ± 2 nm for the 50 h milled sample.

In analysing the lattice strain, an increasing evolution is noticed, and the evolution can be described in a linear way. The equation for this is the following (1):

(1) $$y=0.00178+1.27^{-5}\cdot x.$$

From this equation, an increase in the lattice strain of almost 13% from 30 to 50 h of milling was found. Lattice strain increase versus milling time can be computed using the lattice strain coefficient, given by Equation (2):

(2) $$\alpha =\frac{1}{\varepsilon_{0}}\cdot \frac{∆\varepsilon }{∆t}$$

where *α* is the lattice strain coefficient with milling, *ε*_0_ is the lattice strain intercept, and ∆*ε/*∆*t* is the lattice strain variation with milling time.

The variation rate is found to be 0.00713 (1/h), which is higher compared to the variation rate for the Ni_2_MnSn alloy [43], indicating a higher rate of strain introduction in the powders. This higher strain rate could be responsible for the A_2_ disordered structure obtained by prolonged milling.

**Figure 5:**
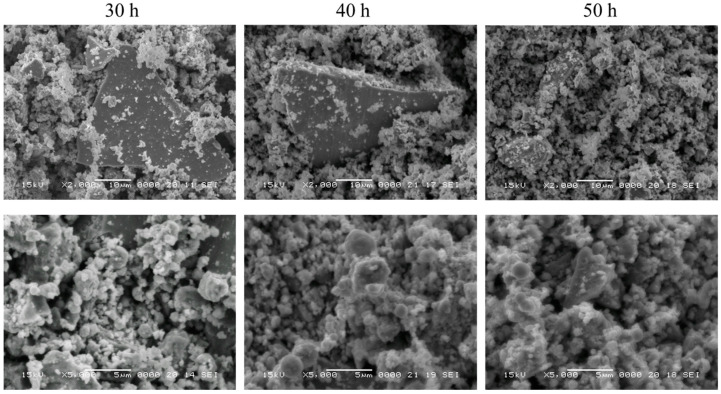
Scanning electron microscopy images of the Fe_50_Mn_35_Sn_15_ powders milled for 30, 40, and 50 h, respectively, recorded at 2000× and 5000×.

Since a multiphase structure was recorded in X-ray diffraction studies, scanning electron microscopy coupled with energy-dispersive studies was conducted. Firstly, aspects regarding the morphology of the powders were investigated, and the recorded images are presented in Figure 5.

The morphology of the milled powders presents two dissimilar types of particles for 30 and 40 h of milling, namely very small-sized particles and large particles. The big particles are as large as 50 µm and have a smooth, clean surface with sharp edges. For the 50 h milled sample, the size of the particles is more homogeneous, with smooth particles of a lesser size. The edges of the larger particles became less sharp at this milling time as compared with lesser milling times. The finer particles agglomerate, a behaviour assigned to cold welding induced by mechanical milling.

**Figure 6:**
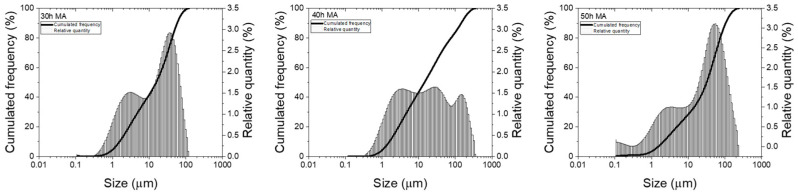
Particle size distributions for 30 to 50 h of milling of the Fe_50_Mn_35_Sn_15_ Heusler alloy.

To understand this particle morphology dynamic, particle size distributions were recorded, and the particle size distributions are presented in Figure 6.

In analysing the particle size distributions for the milled samples, changes are seen. For the 30 h milled sample, the particle size distribution presents two peaks. The first one, recorded for small particle sizes (centred at 3 µm), has a smaller intensity and a wider spread. The second peak, recorded for large particle sizes (centred at 35 µm), has higher intensity and is narrow. This type of distribution suggests that the milled powder has a larger small particle quantity versus the bigger particles, as the SEM images depict.

For milling up to 40 h, the particle size distribution shifts to a three-peak distribution, with almost equal intensities. The first two particle size peaks correspond to the particle sizes observed for the 30 h milled samples, with small variations in size; the third one corresponds to even larger particles (the peak is centred at 150 µm). The appearance of such large particles indicates that intensive welding events occur in the milled powders.

After milling for another 10 h, at 50 h of milling, the three-peak distribution is kept but shifts toward smaller sizes: the first peak at 0.2 µm, the second peak at 3 µm, and the third peak at 54 µm. Again, these recorded particle sizes are in agreement with the SEM images, now showing that the fracture events largely occur in the powders.

The quantification of these results is given in Table 1, where the particle size distribution parameters (D10, D50, D90) are given. The particle size distribution parameters were measured for 30, 40, and 50 h, respectively, to cover the analysed milling range. Larger milling times were not considered since the X-ray diffraction studies indicated no significant evolution of the phases in the considered milling time interval.

**Figure 7:**
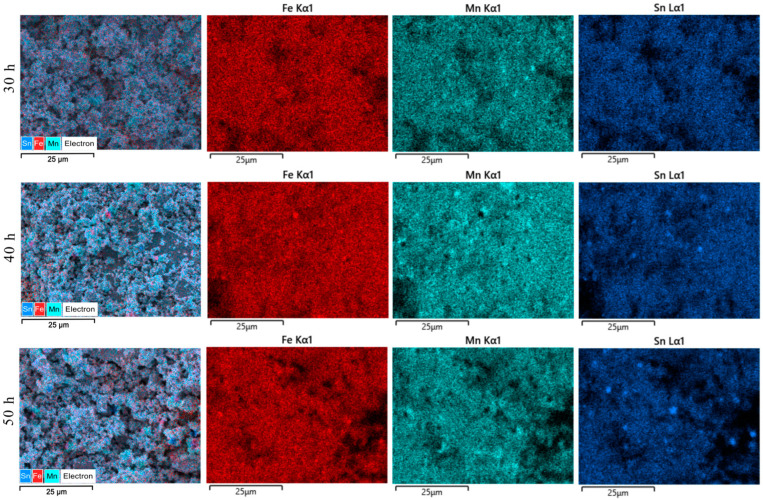
Elemental Fe, Mn, and Sn distribution maps for the Heusler alloy Fe_50_Mn_35_Sn_15_ samples milled for 30 to 50 h, ×2000.

As shown by SEM images and particle size evolutions, an interesting dynamic is in place for Fe_50_Mn_35_Sn_15_ powders milled for times as long as 50 h. To explain this dynamic, the phases contained in the powders must be considered, and most importantly, the size and evolution of the phases, remembering that by X-ray diffraction, a multiphase structure was found. To answer this question, elemental distribution maps were recorded. The EDX maps for the considered milling times are presented in Figure 7.

**Figure 8:**
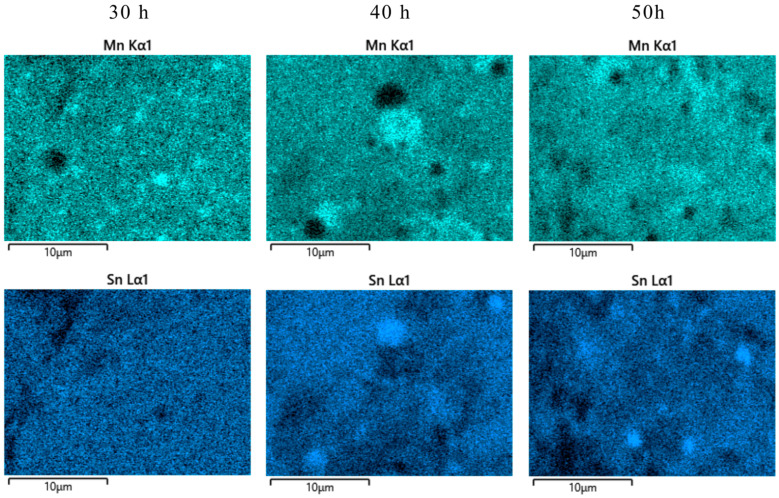
Mn and Sn elemental distribution maps for Fe_50_Mn_35_Sn_15_ Heusler alloy milled for 30, 40, and 50 h at ×5000 magnification.

The distribution maps for the characteristic elements of the Fe_50_Mn_35_Sn_15_ Heusler alloy indicate the existence of clusters for the milled samples. The most homogeneous sample is milled for 30 h, where only some small Mn clusters are seen. The other two elements, Fe and Sn, have a homogeneous distribution. Milling further, the amount of the cluster increases for the Mn element and starts to be a present cluster for Sn and even Fe. To depict the Mn and Sn clustering at an even higher level, the elemental distribution maps for these elements are presented in Figure 8, at higher magnification.

Mn clusters are visible in the 30 h milled sample, but their size seems to be small, with a relatively homogeneous distribution. In the sample milled for 40 h, the amount of Mn clusters is smaller, but their size is larger compared with the 30 h milled sample. By continuing the milling, the Mn cluster size seems to increase and become more irregular. In addition to the Mn clusters, the Sn clusters seem to segregate as the milling is pursued for longer times. The Sn clusters are recorded for a 40 h sample, with a larger volume and in a relatively high amount. For a 50 h milled sample, the Sn clusters are present in a smaller number and size. The Sn clusters are not visible in the 30 h milled sample. This elemental clustering is sustained by the X-ray diffraction studies and phase analysis, where a net increase in Mn phases is computed. In the X_2_MnSn Heusler alloys—obtained by mechanical alloying—Mn clusters can be found [16], but if X is Ni, they disappear for long milling times (after 40 h of milling, according to [43]).

**Figure 9:**
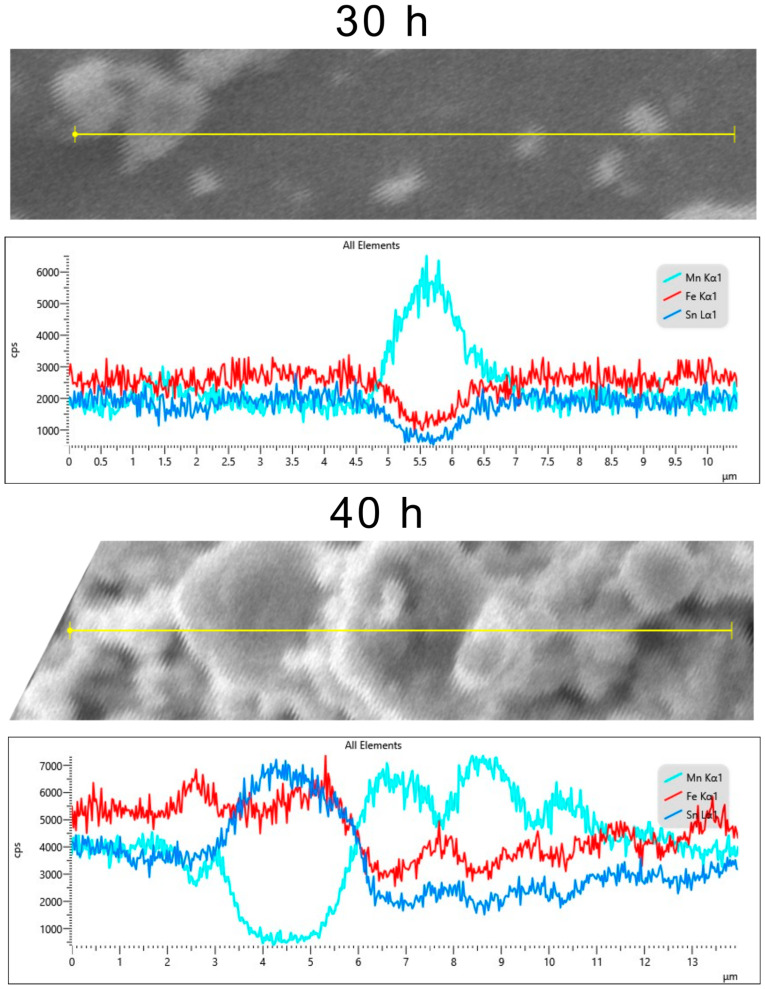

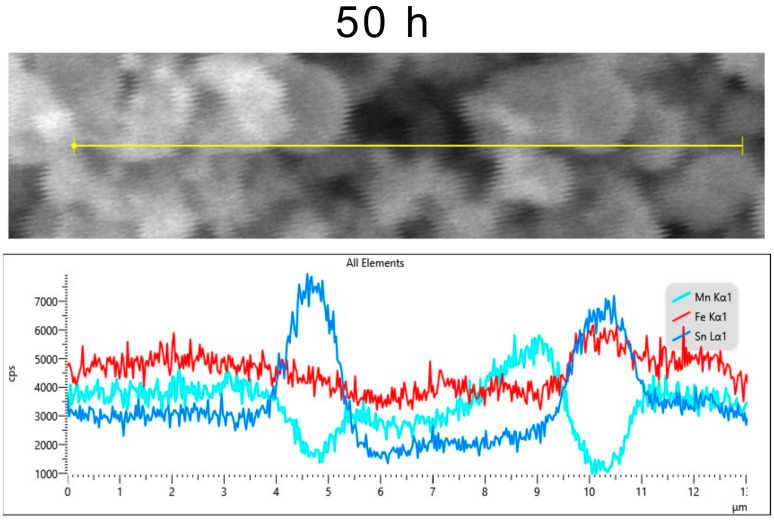
Fe, Mn, and Sn relative amounts over an elemental quantitative acquisition line in the milled Fe_50_Mn_35_Sn_15_ samples.

To completely understand the Mn and Sn clusters, elemental variation over a line in the samples was recorded (Figure 9).

The elemental distribution maps depict well the evolution of the clusters in the milled samples, showing clusters of Mn for the 30 h milled sample, the occurrence of the Sn clusters for the 40 h milled sample, and the stabilisation of Mn and Sn clusters for the 50 h milled sample. Regarding the size of these clusters, the performed measurements indicate that for a 30 h milled sample, its size is almost 1.5 µm, steadily increasing to 1.7 µm at 40 h and to 2.0 µm at 50 h of milling. For the Sn, it is an opposite situation, the clusters of 2.5 µm found at 40 h of milling decrease to 1.5 µm for 50 h milled sample.

**Figure 10:**
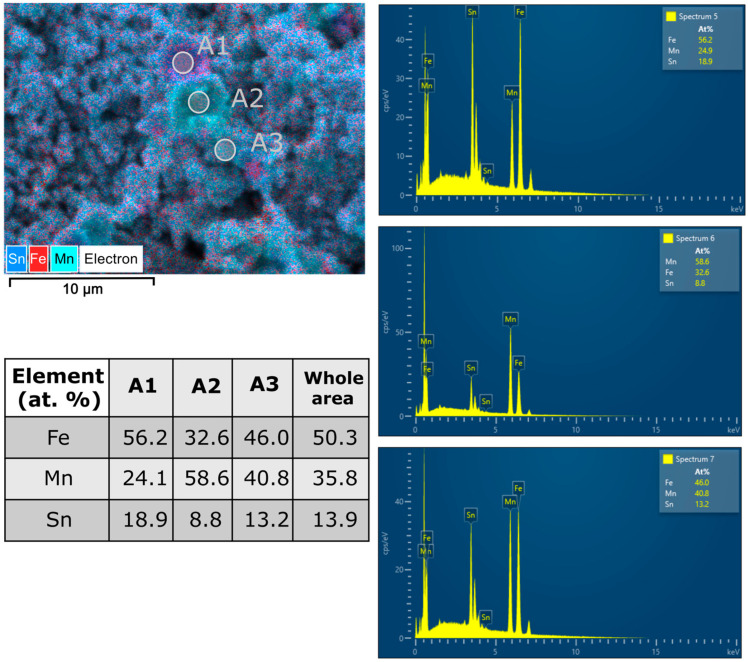
SEM images recorded at 40 h of milling (×5000), showing the superimposed elemental distribution maps, the selected area for analysis (denoted as A1, A2, and A3), the EDX spectra recorded in the selected area, and the table with the chemical composition recorded in the selected area.

**Figure 11:**
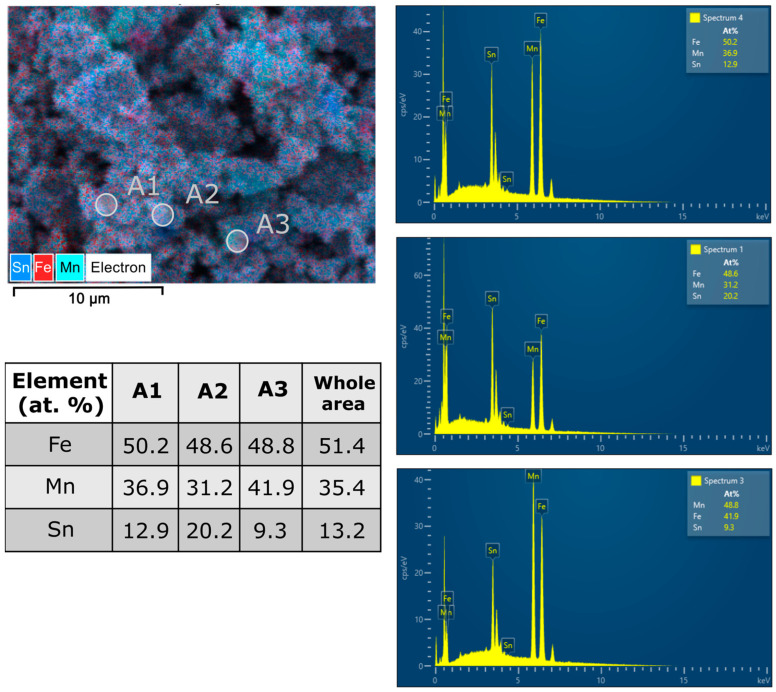
SEM images recorded at 50 h of milling (×5000), showing the superimposed elemental distribution maps, the selected area for analysis (denoted as A1, A2, and A3), the EDX spectra recorded in the selected area, and the table with the chemical composition recorded in the selected area.

Since the samples have multiphase content and the phase formed could play an important role in the physical properties of the milled sample, chemical analysis was performed on selected areas for the long-milled samples. Figure 10 and Figure 11 summarise several composition measurements.

Looking at the chemical composition, the areas analysed show a good stoichiometry for all the milled samples, very close to the composition desired for the Fe_50_Mn_35_Sn_15_ Heusler alloy. Now, looking for the cluster phases, some variation appears, but the identification of the indexed phases by X-ray diffraction is difficult since the signal from a larger area is usually recorded. However, for both milling times (40 and 50 h), a rich Mn area and a rich Sn area are found.

**Figure 12:**
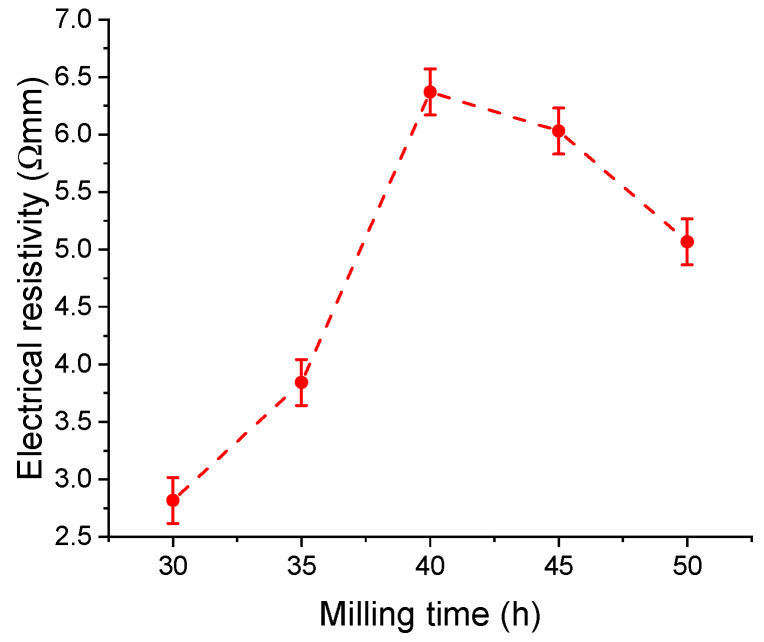
Electrical resistivity of the Fe_50_Mn_35_Sn_15_ Heusler alloy vs. milling time.

From the physical properties of the milled powders, we measured the electrical resistivity with milling time. The evolution of the electrical resistivity with the milling time is presented in Figure 12.

Electrical resistivity increases from 30 to 40 h of milling, from a value of 2.82 × 10^−3^ Ω·m at 30 h to 6.37 × 10^−3^ Ω·m at 40 h. The electrical resistivity increase could be the effect of Sn cluster formation and the formation of Mn-Sn phases. For 40 h of milling, the electrical resistivity of the samples decreases to a value of 5.07 × 10^−3^ Ω·m for the sample milled for 50 h. This resistivity decrease could be the effect of the Sn and Mn cluster homogenisation and Sn cluster size reduction and is probably due to a more uniform distribution of the phases in the sample. As expected, the values are higher than in the cast samples since we performed the measurements on compacted powder samples [18].

## 4. Conclusions

The Fe_50_Mn_35_Sn_15_ Heusler alloy was obtained from elemental powders and its stability was studied in the range of 30 to 50 h of milling. After 30 h of milling, the structure is composed of three phases: a complete disordered Fe_50_Mn_35_Sn_15_ phase (A_2_ structure), with a weighting of 88% of the sample; Mn_3_Sn (10%); and MnSn_2_ (2%). Upon milling, the amount of the Heusler phase decreases to 78%, and the Mn phase amount increases. For the Heusler phase, the mean crystallite size was computed, and it was found that the values slowly decreased with milling time up to 46 ± 2 nm at 50 h of milling. The lattice strain has an increasing behaviour with milling time, and the increase rate was found to be 0.00713 (1/h). The particle size distribution has an increasing tendency for the small and medium particle sizes, indicating that these small particles have a welding tendency as the milling time increases. The elemental distribution of the elements presents a homogeneous distribution for Fe and an increasing number of clusters for Mn. The Mn cluster size increases with milling time from 1.5 (30 h) to 2.0 µm (50 h). After 40 h of milling, Sn clusters start to form with a relatively high diameter (2.5 µm) and are reduced to 1.5 µm at 50 h of milling. Electrical resistivity increases with milling time up to 40 h at a value of 6.37 × 10^−3^ Ω·m, and after Sn cluster formation, a decreasing behaviour is noticed, a value of 5.07 × 10^−3^ Ω·m being measured at 50 h of milling. The nanocrystalline Fe_50_Mn_35_Sn_15_ Heusler alloy, with relatively high electrical resistivity, can be an interesting candidate for magnetic shape memory effect alloys and for theoretical studies that should consider nonequilibrium alloys.

## Figures and Tables

**Table 1:** D10, D50, and D90 particle size distribution parameters for the 30 to 50 h milled Fe_50_Mn_35_Sn_15_ Heusler alloy.

Milling Time (h)	D10	D50	D90
30	1.57	15.55	58.03
40	1.59	14.39	144.49
50	2.10	31.59	104.11

## Data Availability

Data are available upon request.
